# Change in neighborhood socioeconomic status and childhood weight status and body composition from birth to adolescence

**DOI:** 10.1038/s41366-023-01454-7

**Published:** 2024-01-31

**Authors:** Shuang Zhou, Hein Raat, Yueyue You, Susana Santos, Amy van Grieken, Haijun Wang, Junwen Yang-Huang

**Affiliations:** 1 Department of Public Health, Erasmus Medical Center, Rotterdam, The Netherlands; 2 Department of Maternal and Child Health, School of Public Health, Peking University, Beijing, China; 3 The Generation R Study Group, Erasmus MC, University Medical Center, Rotterdam, The Netherlands; 4 Department of Pediatrics, Erasmus MC, University Medical Center, Rotterdam, The Netherlands; 5 EPIUnit - Instituto de Saúde Pública, Universidade do Porto, Rua das Taipas, n° 135, 4050-600 Porto, Portugal; 6 Laboratório para a Investigação Integrativa e Translacional em Saúde Populacional (ITR), Universidade do Porto, Rua das Taipas, n° 135, 4050-600 Porto, Portugal

**Keywords:** Risk factors, Epidemiology, Paediatrics

## Abstract

**Background:**

We aim to assess the associations between the change in neighborhood socioeconomic score (SES) between birth and 6 years and childhood weight status and body composition from 6 to 13 years.

**Methods:**

Data for 3909 children from the Generation R Study, a prospective population-based cohort in the Netherlands were analyzed. The change in neighborhood SES between birth and 6 years was defined as static-high, static-middle, static-low, upward, and downward mobility. Child body mass index (BMI), overweight and obesity (OWOB), fat mass index (FMI) and lean mass index (LMI) were measured at age 6, 10, and 13 years. The associations were explored using generalized estimating equations. The effect modification by child sex was examined.

**Results:**

In total, 19.5% and 18.1% of children were allocated to the upward mobility and downward mobility neighborhood SES group. The associations between the change in neighborhood SES and child weight status and body composition were moderated by child sex (*p* < 0.05). Compared to girls in the static-high group, girls in the static-low group had relatively higher BMI-SDS (β, 95% confidence interval (CI): 0.24, 0.09–0.40) and higher risk of OWOB (RR, 95% CI: 1.98, 1.35–2.91), together with higher FMI-SDS (β, 95% CI: 0.27, 0.14–0.41) and LMI-SDS (β, 95% CI: 0.18, 0.03–0.33). The associations in boys were not significant.

**Conclusions:**

An increased BMI and fat mass, and higher risk of OWOB from 6 to 13 years were evident in girls living in a low-SES neighborhood or moving downward from a high- to a low-SES neighborhood. Support for children and families from low-SES neighborhoods is warranted.

## Introduction

Childhood obesity is a globally recognized public health challenge and a major risk factor for adulthood obesity [1] and other adverse health conditions [2] (i.e., high blood pressure, cardiovascular disease, and metabolic syndrome). Insight into children from which group are at high risk of developing obesity is critical for developing interventions to prevent childhood obesity.

Neighborhood socioeconomic status (SES) plays an important role in the development of childhood obesity [3]. Neighborhoods with low SES often have fewer health-promoting resources, such as physical activity facilities and healthy food retailing [4]. Therefore, living in a socioeconomically disadvantaged neighborhood may be associated with higher risk of obesity through an unbalanced diet and lack of physical activity [4]. Also, neighborhoods with low SES may develop social norms and values that do not promote a healthy lifestyle, which may impact children and then lead to childhood obesity [5].

Existing epidemiological studies linked neighborhood SES at a specific time point to children’s body mass index (BMI) and obesity [6–13], and reported inconsistent results. Some studies in the USA [6–8] and European countries (i.e., Germany [9], Finland [10], and Sweden [11]) reported that low neighborhood SES at birth or at the measurement time of BMI was associated with higher BMI and higher risk of obesity in children aged from 2 to 18 years children. However, other two studies evaluating neighborhood SES at child age 14.5 years in the USA [12] and at child age 4-5 years in Australia [13] reported null associations in adolescents. These inconsistencies might be due to the variations in study areas, the age of the children under study, and the assessment time of neighborhood SES. Since neighborhood SES is dynamic over the life course, studies taking a potential change in neighborhood SES into consideration are needed.

Previous studies has shown that staying in low neighborhood SES or moving downward from high SES to low SES may negatively impact weight status during the whole life [14]. The impact of the change in neighborhood SES on adult weight status has been extensively studied [15–17], but less attention has been given to its impact across childhood and adolescence [15]. The studies on children mostly focused on the change in family SES indicators such as family income and parental educational level [18]. None of these studies evaluated the associations between the change in neighborhood SES and children’s weight status. The life course perspective on health emphasizes that the early years of a child’s life (between birth and age 6 years) represent a sensitive period for the development of weight status [14]. Therefore, examining the associations between the change in neighborhood SES during early life and childhood weight status is essential.

In addition to BMI, measurements on childhood body composition provide further information to distinguish fat mass from lean mass [19]. Compared to BMI, body composition index such as fat mass index (FMI) and lean mass index (LMI) is better indicator to predict the risk of diabetes and cardiovascular disease [20]. Therefore, studying the risk factors of childhood body composition is able to provide additional value for later health. A recent review has showed the effect of family SES on children’s body composition [21], but less attention has been given to the associations between neighborhood SES and children’s body composition. To our knowledge, only one study in the UK has reported that a disadvantaged neighborhood SES is associated with overall higher FMI from child age 7 to 17 years [22].

This study aimed to examine the associations between the change in neighborhood SES between birth and 6 years old and children’s weight status (BMI-SDS and OWOB) and body composition (FMI-SDS and LMI-SDS) from 6 to 13 years old, using a longitudinal population-based design. In addition, we evaluated to what extent the associations were modified by child sex.

## Methods

### Study design and participants

This study was embedded in the Generation R study, which is a population-based prospective cohort from fetal life onwards in Rotterdam, the Netherlands [23]. Pregnant women with an expected delivery date between April 2002 and January 2006 were invited into the cohort during their prenatal visit. As shown in the flowchart (Supplementary Fig. S1), a total of 8305 children participated in the study at age 6 years. Children without data on residential zip codes at birth or 6 years were excluded (*n* = 2151). To avoid clustering of data, twin children (*n* = 161), or the second (*n* = 383) and third (*n* = 7) children of the same mother were excluded. Children with missing measurements of BMI in more than one time point at 6, 10, and 13 years were excluded for the analyses for weight status (BMI-SDS and OWOB) (*n* = 1694). Children with missing measurement of body composition indicators (either FMI or LMI) in more than one time point at 6, 10, and 13 years were excluded for the analyses for FMI-SDS and LMI-SDS (*n* = 1767). Finally, a total of 3909 children were included in the analyses for BMI-SDS/OWOB (*n* = 3909) and FMI/LMI-SDS (*n* = 3836), respectively. The Generation R Study was approved by the Medical Ethical Committee of the Erasmus Medical Center, Rotterdam (MEC 217.595/2002/202). Written informed consent was obtained from all participants.

### Neighborhood socioeconomic status

The neighborhood zip code was obtained by parent-reported questionnaires at birth (2002–2006) and at child age 6 years (2008–2012). Information on neighborhood SES was obtained from the Netherlands Institute of Social Research (SCP) [24]. Neighborhood SES scores were computed by SCP in 2002, 2006, 2010, 2014, 2016, and 2017 with principal component analysis based on the mean resident income, percentage of low resident incomes, percentage of low educated residents, and percentage of unemployed residents in a neighborhood [24]. The nearest data available on neighborhood SES scores were matched to the neighborhood zip code at birth (2006), at child age 6 years (2010), and at child age 13 years (2017). Participants were categorized in three groups according to their neighborhood SES scores: low neighborhood SES (1^st^ tertiles), middle neighborhood SES (2^nd^ tertiles), and high neighborhood SES (3^rd^ tertiles).

The change in neighborhood SES was classified into five categories based on whether the neighborhood SES group changed upward or downward or stayed static comparing birth and 6 years: static-high group (high to high), static-middle group (middle to middle), static-low group (low to low), upward mobility group (low to middle, low to high, and middle to high), and downward mobility group (high to middle, high to low, and middle to low).

### Weight status and body composition

Child height and weight were measured by well-trained staff at child age 6, 10, and 13 years, with standardized procedures [25] and calibrated instruments. Height was measured in a standing position to the nearest millimeter with a Harpenden stadiometer (Holtain Ltd, Dyfed, The United Kingdom). Weight was measured with light clothes to the nearest gram using a mechanical personal scale (SECA, Almere, The Netherlands). BMI was calculated as weight divided by height squared (kg/m^2^). Age- and sex-adjusted BMI standard deviation scores (BMI-SDS) were obtained using Dutch reference growth charts [26] in the Growth Analyzer program (http://www.growthanalyser.org). Children were categorized as overweight/obesity (OWOB) or normal weight according to cutoff points from the International Obesity Task Force [27].

Children’s body composition was measured at child age 6, 10, and 13 years using a DXA scanner (iDXA, GE-Lunar, 2008, Madison, WI, USA) and analyzed with the enCORE software, version 12.6 (GE-Healthcare). FM and LM were log-transformed by natural logs and regressed on log-transformed height [28], which has been described in detail previously [29]. Briefly, we got the regression slopes at each age, which were used as the power in the following calculation: FM (or LM /height^^(regression slope)^). We calculated FMI with fat mass (kg)/height^3^ (m^3^) at 6 years, fat mass (kg)/height^4^ (m^4^) at 10 years, and fat mass (kg)/height (m) at 13 years, respectively. We calculated LMI with lean mass (kg)/height^2^ (m^2^) at 6 years, lean mass (kg)/ height^2^ (m^2^) at 10 years, and lean mass (kg)/ height^3^ (m^3^) at 13 years, respectively. FMI-SDS and LMI-SDS were calculated on the basis of the total Generation R study population with body composition data at 6, 10, and 13 years old, respectively.

### Covariates

Covariates were chosen based on previous literature [22] and we drew a directed acyclic graph (Supplementary Fig. S2), including maternal age, maternal educational level, net household income, child ethnic background, sex, and birth weight. Maternal age was obtained by a parent-reported questionnaire at enrollment. The highest completed maternal educational level and net household income were obtained using parent-reported questionnaire at child age 6 years. Information on children’s sex and birth weight was obtained from medical records at birth. Child ethnic background was classified into three categories according to the countries of birth of the parents: Dutch, other Western, and non-Western (Indonesian, Cape Verdean, Moroccan, Dutch Antilles, Surinamese, Turkish, African, American non-western, and Asian non-western). If both parents were born in the Netherlands, child ethnic background was Dutch. If one of the parents was born outside the Netherlands, child ethnic background was determined by this country. If both parents were born outside the Netherlands, child ethnic background was determined by the country of birth of the mother. Maternal educational level was categorized into low (no education, primary school, lower vocational training, intermediate general school, or 3 years or less general secondary school), middle (>3 years general secondary school, intermediate vocational training), and high (higher vocational training, university or Ph.D. degree) [30]. Net household income was categorized into low (<€2400/month), middle (€2400–€4000/month), and high (≥€4000/month) [31].

### Statistical analyses

Descriptive analysis of population characteristics was performed. Characteristics of boys and girls according to the groups with different change in neighborhood SES were compared using t-test (or ANOVA test) and chi-square test.

Generalized estimating equations (GEE) were used to analyze the association of the change in neighborhood SES and children’s weight status (BMI-SDS and OWOB) and body composition (FMI-SDS and LMI-SDS) from 6 to 13 years old [32]. A First-order auto regressive correlation structure was used to take into account children’s repeated measurements of weight status. Two sets of models were created. Model 1 was a crude model without adjusting for any potential covariates. Model 2 was adjusted for potential covariates, including maternal age, maternal educational level, net household income, child ethnic background, and birth weight. In the models of body composition, we additionally adjusted for child sex and age. In all models, the Beta (β) or risk ratios (RR) and their 95% confidence interval (95%CI) were reported using the static-high group as the reference group. Previous literature has found different effect of neighborhood SES on childhood weight status and body composition in boys and girls [21], thus the effect modification by child sex was evaluated by including the interaction term between the change in neighborhood SES and child sex in the model. In case of significant interaction effect (*p* < 0.05), stratified analyses were performed.

A multiple imputation procedure based on Multivariate Imputation by Chained Equations was used to impute missing values in the covariates. Five imputed data sets were generated using the MICE package (version 3.14.0) in statistical software R. Pooled effect estimates (RRs and beta coefficients) and the 95% CIs from these five imputed datasets were reported.

Sensitivity analysis was performed. Firstly, children were categorized into two groups: children who relocated and children who did not move based on whether the residential address at birth and 6 years were the same or not. The association between the change in neighborhood SES and children’s growth trajectory was then assessed stratified by whether children relocated or not. Secondly, we explored the association between the change in neighborhood SES and child weight status and body composition change adjusting for the change in neighborhood SES between 6 and 13 years old. Thirdly, we adjusted for children’s psychosocial health status evaluated using the Child Behavior Checklist (CBCL) [33].

All statistical analyses were performed with the statistical software R 4.1.3 (R Core Team 2020). A two-sided *P* value < 0.05 was considered as statistically significant.

## Results

### Characteristics of the participants

The characteristics of the study population are presented in Table 1. Of the 3909 children in the cohort, 24.8%, 16.5%, and 21.2% of the children were in the static-high, static-middle, and static-low neighborhood SES group; 19.5% of the children were in the upward mobility group and 18.1% of the children were in the downward mobility group. In total, 39.2% of children relocated between birth and 6 years. The percentages of children who were OWOB at age 6, 10, and 13 years are 17.9%, 20.3%, and 18.0% respectively. At age 10 and 13 years, boys had lower BMI than girls (both *p* < 0.05). At age 6 and 10 years, the percentage of OWOB was lower in boys (15.1% and 18.0%, respectively) than in girls (20.7% and 22.6%, respectively). Boys had lower FMI but higher LMI than girls at 6, 10, and 13 years old (all *p* < 0.05). There was no significant difference in maternal age, maternal educational level, net household income, child ethnic background, neighborhood SES, and BMI-SDS between boys and girls.

**Table 1:** Characteristics of participants in the study (*N* = 3909).

	Total *N* = 3909	Boy *N* = 1956	Girl *N* = 1953	*P*-value^b^
Family characteristics				
Maternal age at enrollment (years), mean (SD)	30.9 (5.2)	31.0 (5.2)	30.8 (5.2)	0.34
Maternal education level, *N* (%)^a^				0.46
High	1806 (54.2)	920 (55.1)	886 (53.2)	
Middle	1081 (32.4)	525 (31.4)	556 (33.4)	
Low	448 (13.4)	226 (13.5)	222 (13.3)	
Net Household income, *N* (%)^a^				0.96
<2400	1066 (33.7)	541 (33.9)	525 (33.5)	
2400~4000	1115 (35.2)	559 (35.0)	556 (35.5)	
≥4000	983 (31.1)	497 (31.1)	486 (31.0)	
Neighborhood socioeconomic status				
Neighborhood SES at birth, *N* (%)^a^				0.93
High	1561 (39.9)	779 (39.8)	782 (40.0)	
Middle	1053 (26.9)	532 (27.2)	521 (26.7)	
Low	1295 (33.1)	645 (33.0)	650 (33.3)	
Neighborhood SES at child age 6 years old, *N* (%)^a^				0.48
High	1363 (34.9)	698 (35.7)	665 (34.1)	
Middle	1566 (40.1)	767 (39.2)	799 (40.9)	
Low	980 (25.1)	491 (25.1)	489 (25.0)	
Neighborhood SES mobility from birth to 6 years old, *N* (%)^a^				0.75
Static-high group	970 (24.8)	501 (25.6)	469 (24.0)	
Static-middle group	645 (16.5)	323 (16.5)	322 (16.5)	
Static-low group	827 (21.2)	414 (21.2)	413 (21.1)	
Upward mobility group	761 (19.5)	377 (19.3)	384 (19.7)	
Downward mobility group	706 (18.1)	341 (17.4)	365 (18.7)	
Relocation, *N* (%)^a^	1534 (39.2)	785 (40.1)	749 (38.4)	0.25
Child characteristics				
Birth weight (grams), mean (SD)	3418.0 (555.3)	3489.5 (569.9)	3346.4 (530.8)	**<0.001**
Child ethnic background, *N* (%)^a^				0.32
Dutch	2070 (54.3)	1034 (54.1)	1036 (54.5)	
Other western	329 (8.6)	154 (8.1)	175 (9.2)	
Non-western	1414 (37.1)	723 (37.8)	691 (36.3)	
Child weight status				
BMI, mean (SD)				
At 6 years	16.2 (1.9)	16.2 (1.7)	16.3 (2.1)	0.36
At 10 years	17.7 (2.9)	17.6 (2.7)	17.9 (3.1)	**<0.01**
At 13 years	20.1 (3.7)	19.6 (3.4)	20.5 (3.8)	**<0.001**
Overweight/obesity (yes), *N* (%)^a^				
At 6 years	680 (17.9)	289 (15.1)	391 (20.7)	**<0.001**
At 10 years	751 (20.3)	333 (18.0)	418 (22.6)	**<0.01**
At 13 years	579 (18.0)	279 (17.5)	300 (18.6)	0.73
Child body composition				
FMI, mean (SD)				
At 6 years	3.4 (1.1)	3.1 (1.0)	3.8 (1.2)	**<0.001**
At 10 years	2.5 (1.1)	2.3 (1.0)	2.7 (1.1)	**<0.001**
At 13 years	8.9 (4.3)	7.7 (4.0)	10.0 (4.3)	**<0.001**
LMI, mean (SD)				
At 6 years	11.4 (0.9)	11.7 (0.8)	11.1 (0.9)	**<0.001**
At 10 years	12.0 (1.1)	12.3 (1.0)	11.7 (1.1)	**<0.001**
At 13 years	8.4 (0.9)	8.5 (0.9)	8.3 (0.9)	**<0.001**

Missing number: maternal educational level: 574 (14.7%), household income: 745 (19.0%), birth weight: 3 (0.1%), ethnic background: 96 (2.5%), BMI at 6 years: 106 (2.7%), BMI at 10 years: 191 (4.9%), BMI at 13 years: 701 (17.9%), FMI at 6 years: 206 (5.3%), FMI at 10 years: 233 (6.0%), FMI at 13 years: 885 (22.6%), LMI at 6 years: 206 (5.3%), LMI at 10 years: 233 (6.0%), LMI at 13 years: 885 (22.6%), Overweight/obesity at 6 years: 111 (2.8%), Overweight/obesity at 10 years: 203 (5.2%), Overweight/obesity at 13 years: 701 (17.9%).

*BMI* body mass index, *FMI* fat mass index, *LMI* lean mass index.

^a^The percentage for categorical variables is valid percentage.

^b^T-test was applied for continuous variables and chi-square test for categorical variables to test the differences between boys and girls.

The significant results are in bold in the table (*P* < 0.01).

As shown in Supplementary Table S1, compared with children included in the study, children not included were more likely to have a mother with younger age (*p* < 0.001) and higher educational level (*p* < 0.001), come from high-income household (*p* < 0.001), and have a Dutch ethnic background (*p* < 0.001).

The distribution of maternal education level, household income, and child ethnic background varied in the five groups of the change in neighborhood SES (all *p* < 0.001, Table 2). More than 70% of children had a mother who finished a high educational level in the static-high group while only 29.6% in the static-low group. In the static-high group, 55.6% of children came from high-income household families and 80.2% had a Dutch ethnic background, while in the static-low group the percentages were 8.8% and 23.1%, respectively. Children in the static-high group had the lowest BMI and lowest risk of OWOB (*p* < 0.001) together with the lowest FMI-SDS (*p* < 0.001), LMI-SDS (*p* < 0.05, except at age 10 years) at age 6,10, and 13 years.

**Table 2:** Characteristics of the participants according to the change in neighborhood socioeconomic status (*N* = 3909).

	Static-high group *N* = 970	Static-middle group *N* = 645	Static-low group *N* = 827	Upward mobility group *N* = 761	Downward mobility group *N* = 706	*P*-value^b^
Family characteristics						
Maternal age at enrollment (years), mean (SD)	32.8 (3.9)	30.8 (5.0)	29.2 (5.8)	30.3 (5.4)	30.9 (4.9)	**<0.001**
Maternal education level, *N* (%)^a^						**<0.001**
High	667 (72.0)	284 (51.9)	180 (29.6)	362 (58.6)	313 (49.3)	
Middle	220 (23.7)	185 (33.8)	250 (41.1)	182 (29.4)	244 (38.4)	
Low	40 (4.3)	78 (14.3)	178 (29.3)	74 (12.0)	78 (12.3)	
Household income, *N* (%)^a^						**<0.001**
<2400	88 (10.2)	208 (40.5)	365 (63.0)	233 (39.2)	172 (28.2)	
2400–4000	296 (34.2)	173 (33.7)	163 (28.2)	191 (32.1)	292 (47.8)	
≥4000	481 (55.6)	133 (25.9)	51 (8.8)	171 (28.7)	147 (24.1)	
Child characteristics						
Sex						0.75
Boy	501 (51.6)	323 (50.1)	414 (50.1)	377 (49.5)	341 (48.3)	
Girl	469 (48.4)	322 (49.9)	413 (49.9)	384 (50.5)	365 (51.7)	
Birth weight (grams), mean (SD)	3509.5 (530.4)	3407.9 (569.0)	3346.1 (553.0)	3396.6 (588.9)	3408.6 (525.7)	**<0.001**
Child ethnic background, *N* (%)^a^						**<0.001**
Dutch	776 (80.2)	303 (48.2)	181 (23.1)	357 (48.4)	453 (65.2)	
Other western	80 (8.3)	59 (9.4)	53 (6.8)	83 (11.2)	54 (7.8)	
Non-western	112 (11.6)	266 (42.4)	550 (70.2)	298 (40.4)	188 (27.1)	
Child weight status						
BMI, mean (SD)						
At 6 years	15.8 (1.4)	16.3 (1.9)	16.7 (2.3)	16.2 (1.9)	16.1 (1.8)	**<0.001**
At 10 years	17.0 (2.1)	17.8 (3.0)	18.6 (3.5)	17.7 (2.9)	17.6 (2.8)	**<0.001**
At 13 years	19.2 (2.7)	19.9 (3.4)	21.3 (4.5)	20.2 (3.7)	20.0 (3.6)	**<0.001**
FMI, mean (SD)						
At 6 years	3.1 (0.8)	3.5 (1.1)	3.7 (1.4)	3.4 (1.1)	3.4 (1.0)	**<0.001**
At 10 years	2.2 (0.8)	2.6 (1.1)	2.9 (1.3)	2.5 (1.0)	2.5 (1.0)	**<0.001**
At 13 years	7.8 (3.2)	8.7 (4.1)	10.3 (5.2)	9.0 (4.4)	8.9 (4.2)	**<0.001**
LMI, mean (SD)						
At 6 years	11.4 (0.8)	11.4 (0.9)	11.5 (1.0)	11.4 (0.9)	11.4 (0.9)	0.08
At 10 years	11.9 (0.9)	12.0 (1.1)	12.1 (1.2)	12.0 (1.1)	12.0 (1.1)	**<0.001**
At 13 years	8.2 (0.8)	8.4 (0.9)	8.6 (1.0)	8.4 (0.9)	8.4 (0.9)	**<0.001**
Overweight or obesity (yes), *N* (%)^a^						
At 6 years	90 (9.6)	125 (19.7)	202 (25.3)	138 (18.6)	125 (18.2)	**<0.001**
At 10 years	102 (10.8)	135 (22.2)	241 (31.5)	137 (19.0)	136 (20.3)	**<0.001**
At 13 years	85 (10.4)	88 (16.8)	177 (27.7)	114 (17.9)	115 (19.5)	**<0.001**

Missing number: maternal educational level: 574(14.7%), household income: 745(19.0%), birth weight: 3(0.1%), ethnic background: 96(2.5%), BMI-SDS at 6 years:106(2.7%), BMI-SDS at 10 years: 191(4.9%), BMI-SDS at 13 years: 701(17.9%), FMI-SDS at 6 years: 206(5.3%), FMI-SDS at 10 years: 233(6.0%), FMI-SDS at 13 years: 885(22.6%), LMI-SDS at 6 years: 206(5.3%), LMI-SDS at 10 years: 233(6.0%), LMI-SDS at 13 years: 885(22.6%), Overweight/obesity at 6 years: 111(2.8%),

Overweight/obesity at 10 years: 203(5.2%), Overweight/obesity at 13 years: 701(17.9%),

*BMI* body mass index, *FMI* fat mass index, *LMI* lean mass index.

^a^The percentage for categorical variables is valid percentage.

^b^The t-test for continuous variables and chi-square test for categorical variables were used to test the differences among different neighborhood SES mobility groups.

The significant results are in bold in the table (*P* < 0.001).

### Associations between the change in neighborhood SES and childhood weight status and body composition

The adjusted associations between the change in neighborhood SES between birth and 6 years and childhood weight status and body composition from 6 to 13 years were shown in Table 3. The interaction analysis indicated that the associations were modified by sex (*P* < 0.05). Compared to girls in the static-high group, girls in the static-low group had relatively higher BMI-SDS (β, 95% CI: 0.24, 0.09–0.40). Compared to girls in the static-high group, girls had higher risk of OWOB from 6 to 13 years in the static-middle (RR, 95% CI: 1.54, 1.06–2.24), static-low (1.98, 1.35–2.91) and downward mobility group (1.66, 1.15–2.38). Compared to girls in the static-high group, girls in the static-low group had relatively higher FMI-SDS (β, 95% CI: 0.27, 0.13–0.41) and LMI-SDS (0.18, 0.04–0.33) from 6 to 13 years old. In boys, these associations were not significant. The crude results were shown in Supplementary Table S2.

**Table 3:** Associations between the change in neighborhood SES between birth and 6 years and childhood weight status and body composition from 6 to 13 years.

	Boy			Girl			*P*_*interaction*_^b^
	*n*	β/RR (95% CI)^a^	*P*-value	*n*	β/RR (95% CI)^a^	*P*-value	
Weight status from 6 to 13 years old							
BMI-SDS							
Static-high group	501	Ref	Ref	469	Ref	Ref	
Static-middle group	323	0.00 (−0.14, 0.13)	0.96	322	0.09 (−0.04, 0.22)	0.17	0.17
Static-low group	414	0.04 (−0.11, 0.19)	0.59	413	**0.24 (0.09, 0.40)**	**<0.01**	**<0.01**
Upward mobility group	377	0.02 (−0.11, 0.14)	0.79	384	0.05 (−0.08, 0.17)	0.47	0.42
Downward mobility group	341	0.10 (−0.03, 0.23)	0.15	365	0.01 (−0.12, 0.14)	0.88	0.53
OWOB							
Static-high group	501	Ref	Ref	469	Ref	Ref	
Static-middle group	323	1.23 (0.84, 1.78)	0.29	322	**1.54 (1.06, 2.24)**	**0.02**	0.26
Static-low group	414	1.06 (0.71, 1.56)	0.78	413	**1.98 (1.35, 2.91)**	**<0.001**	**<0.01**
Upward mobility group	377	1.15 (0.80, 1.65)	0.44	384	1.30 (0.89, 1.88)	0.17	0.44
Downward mobility group	341	1.35 (0.93, 1.97)	0.11	365	**1.66 (1.15, 2.38)**	**<0.01**	0.29
Body composition from 6 to 13 years old							
FMI-SDS							
Static-high group	492	Ref	Ref	458	Ref	Ref	
Static-middle group	318	0.07 (−0.06, 0.20)	0.29	318	0.12 (0.00, 0.23)	0.05	0.34
Static-low group	407	0.12 (−0.02, 0.25)	0.10	412	**0.27 (0.14, 0.41)**	**<0.01**	**0.01**
Upward mobility group	364	0.05 (−0.07, 0.17)	0.41	375	0.07 (−0.04, 0.18)	0.23	0.45
Downward mobility group	337	0.10 (−0.02, 0.22)	0.12	355	0.05 (−0.05, 0.16)	0.33	0.78
LMI-SDS							
Static-high group	492	Ref	Ref	458	Ref	Ref	
Static-middle group	318	−0.05 (−0.17, 0.07)	0.42	318	0.10 (−0.03, 0.23)	0.13	0.06
Static-low group	407	−0.05 (−0.18, 0.08)	0.49	412	**0.18 (0.03, 0.33)**	**0.02**	**<0.01**
Upward mobility group	364	0.00 (−0.11, 0.12)	0.94	375	0.04 (−0.08, 0.16)	0.52	0.51
Downward mobility group	337	0.08 (−0.04, 0.19)	0.21	355	0.03 (−0.10, 0.15)	0.65	0.66

^a^In the model of BMI-SDS and OWOB, adjusted for maternal age, maternal educational level, family income, birth weight and ethnicity. In the model of FMI-SDS and LMI-SDS, child age was additionally adjusted.

^b^Wald test was used to test the interaction effect of neighborhood SES mobility and child sex.

### Sensitivity analysis

Supplementary Table S3 showed the adjusted associations between the change in neighborhood SES and childhood weight status and body composition stratified by whether children relocated between birth and 6 years. In the children who did not move to a new address, children in the static-low group had relatively higher BMI-SDS (β, 95% CI: 0.20, 0.07–0.33) compared to children in the static-high group. Compared to children in the static-high group, children had higher risk of OWOB from 6 to 13 years in the static-middle (RR, 95% CI: 1.64, 1.20–2.24), static-low (1.64, 1.18–2.30) and downward mobility group (1.70, 1.22–2.37). Compared to children in the static-high group, children had relatively higher FMI-SDS from 6 to 13 years old in the static-middle group (β, 95% CI: 0.14, 0.04–0.24), static-low group (0.25, 0.13–0.36), and upward mobility group (0.13, 0.01–0.26). In the subgroup of children who relocated, no significant association was observed between the change in neighborhood SES and childhood weight status and body composition.

After additionally adjusting for the change in neighborhood SES between 6 and 13 years old, the associations between the change in neighborhood SES and childhood weight status and body composition were in the same direction as the main analyses, while the effect estimates coefficients (β/RRs) were not statistically significant (Supplementary Table S4).

When we additionally adjusted for child psychosocial health status, the results were comparable with the main analyses (Supplementary Table S5).

## Discussion

This study showed that among girls, staying in a lower-SES neighborhood or moving downward from a higher- to lower-SES neighborhood was significantly associated with relatively higher BMI-SDS and higher risk of OWOB, together with higher FMI-SDS and LMI-SDS from 6 to 13 years. The associations in boys had the same direction but did not reach significance.

Previous studies indicated that disadvantaged neighborhood SES at one time point was associated with increased BMI and higher risk of OWOB in children [6–11, 22]. Our findings add to the literature by showing the effect of change in neighborhood SES on several indicators of weight status and body composition. Namely girls living in a low-SES neighborhood or moving downwards from a high- to a low-SES neighborhood, had a relatively higher BMI and higher risk of OWOB from 6 to 13 years old. Previous studies examined the effect on the change of neighborhood SES were mainly conducted in adults. The results were consistent with ours showing that a negative change in neighborhood SES can be associated with increased weight in later years [16, 17]. However, these findings should be interpreted with caution. When accounting for the change in neighborhood SES between 6 and 13 years, these were not statistically significant but in the same direction as the main results. More studies are needed to study the impact of neighborhood SES change on weight status at early ages.

Previous studies on the association of change in family SES (i.e., parental education level) and child OWOB showed that attaining a high education level after birth for both mothers and fathers can be beneficial to attenuate the risk of the child having OWOB at child age six and ten years [34]. Our study contributes to the previous findings that neighborhood SES may have a comprehensive impact on child health through the built environment, health intervention programs, and social norms in the neighborhood [4].

In addition to BMI and OWOB, this study establishes knowledge of how the change in neighborhood SES may influence child body composition. The findings on the association between the change in neighborhood SES with FMI-SDS and LMI-SDS are in line with the observed increases in BMI and higher risk in OWOB in our study. One study in the UK investigated the associations between neighborhood SES at 9 months and children’s body composition trajectory from 7 to 17 years old [22]. This study showed that disadvantaged neighborhood SES was associated with higher FMI, which was consistent with our finding. Body composition (i.e., FMI and LMI) is reported to be better indicator to predict the risk of metabolic disease than BMI. Studying the association between the change in neighborhood SES and the child body composition over time offers more insight into the development of child adiposity and the prediction of long-term health. Therewith, it may offer insights into who and when interventions with regard to obesity can be offered timely. As the first study on the effect of the change in neighborhood SES on child weight status and body composition, our findings provide evidence for public supports to improve the neighborhood SES to decrease the risk of unhealthy weight and non-ideal body composition. Neighborhoods with low SES often have fewer health-promoting built environment characteristics, such as physical activity facilities and healthy food retailing [4]. Strong evidence was found for the association between more built environment characteristics supportive of walking (neighborhood walkability and availability) and accessibility of parks and playgrounds with lower prevalence of childhood obesity [35]. Built environment thus played a key factor in the association between neighborhood SES and childhood obesity. Previous studies have demonstrated that building walking trails and play area in the neighborhood, increasing the amount of physical activity equipment, improving the existing equipment resources, increasing the number of groceries selling healthy food can help to prevent childhood obesity [36, 37]. Moreover, implementing physical activity programs (i.e., youth sports clubs and adult yoga classes) and nutrition-related programs (i.e., cooking and nutrition education classes) in the neighborhood are also beneficial for children in risk [36, 37]. Therefore, intervention programs regarding the built environment and physical and dietary behavior shall be developed to support and encourage healthy dietary and physical activity behaviors in deprived neighborhoods.

Consistent with previous studies [21], the change in neighborhood SES had different effects on children’s growth trajectory in boys and girls. In our study, girls living in or relocating to a deprived neighborhood in early childhood had relatively higher weight, while we couldn’t find the significant effect in boys. Families in low-SES neighborhoods were more easily to be influenced by the shared unhealthy norms and values in the neighborhood (e.g. less exercise and having more fast food) [5]. Compared to girls, boys were reported to be less influenced by the sharing norms which were adopted by their parents. Thus, this might be the explanation for the null associations in boys. Besides, children in low-SES neighborhoods might be exposed to more and continuous psychosocial stressors than children in high-SES neighborhoods. Younger children had few abilities to cope with the stressors [38]. Different coping strategies, such as stress eating and increasing screen time might be the possible reason to explain the findings in boys and girls [38]. When we additionally adjusted for children’s psychosocial health status in the models, the associations between the change in neighborhood SES and children’s weight status and body composition remained consistent with the main results, which indicated that children’s psychosocial health status might only partly mediate the associations. Further studies are needed to clarify the mechanisms.

The change in neighborhood SES may be related to either children’s relocation or the change in neighborhood conditions itself. In our study, we found a consistent association between staying in a lower-SES neighborhood and child weight status and body composition in children who didn’t move to another place. We additionally observed that in children without residential mobility, upward mobility was associated with higher fat mass. In children who relocated in early ages, no association was found between the change in neighborhood SES and child weight status and body composition. Considering that existing studies exploring the difference between movers and non-movers on the associations of change in neighborhood SES and childhood growth remains sparse, we were unable to compare our results with other previous findings. However, previous study in adults has reported that the associations between the change in neighborhood SES and weight change were more pronounced for non-movers than movers [39], which can partly support our findings. When children relocated to another neighborhood, they may not adapt to the new environment and may not be familiar with the neighborhood resource, which could hinder them using these resources to protect them from obesity [40]. However, these findings should be interpreted with caution due to the limited sample size. Future studies exploring the effect of the change in neighborhood SES in children whether relocated or not are warranted in large study sample size to provide more insights into the effect of neighborhood improvement on the child growth trajectory.

### Methodological considerations

There are several strengths of this study. First, the study design of a well-conducted prospective birth cohort with a large sample size supports the validity of our results. Besides, we repeatedly evaluated neighborhood SES at birth and child age at 6 years, using a standardized and comprehensive indicator based on objective measures of resident income, unemployment rate, and educational level with high geographical resolution. Moreover, the prospective study design with standardized weight status and body composition measurements at three different time points provides reliable data from childhood to early adolescence.

Nevertheless, several limitations should be considered. First, the possibility of residual confounders cannot be precluded. Second, variables on neighborhood built environment (e.g., physical activity facilities, food retailing, walkability and cyclability) were not included in the main analyses. These variables may be considered as mediators, explaining the associations between the change of neighborhood SES and child weight status and body composition. Future studies should explore specific pathways related to child weight status and body composition between subgroups with different changes in neighborhood SES. Third, the indicator of neighborhood SES only reflects the level of income, education, and employment. Other domains of neighborhood SES should be considered in the future.

## Conclusion

In conclusion, an increased BMI and fat mass, and higher risk of OWOB from 6 to 13 years were evident in girls living in a low-SES neighborhood or moving downward from a high- to a low-SES neighborhood. These findings support the need of overweight prevention policies to address for children from lower socioeconomic status neighborhoods.

### Supplementary information

Supplementary file

## Data Availability

The data underlying this article will be shared on reasonable request to the corresponding author.
