# Supplementary material for: Change in neighborhood socioeconomic status and childhood weight status and body composition from birth to adolescence
Source: Int J Obes (Lond). 2024 Jan 31;48(5):646–53. doi: 10.1038/s41366-023-01454-7 (PMC11058568; doi:10.1038/s41366-023-01454-7)
Supplement: Supplementary file 1 — Supplementary file [file 41366_2023_1454_MOESM1_ESM.docx]

**Change in neighborhood socioeconomic status and childhood weight status and body composition from birth to adolescence**

**
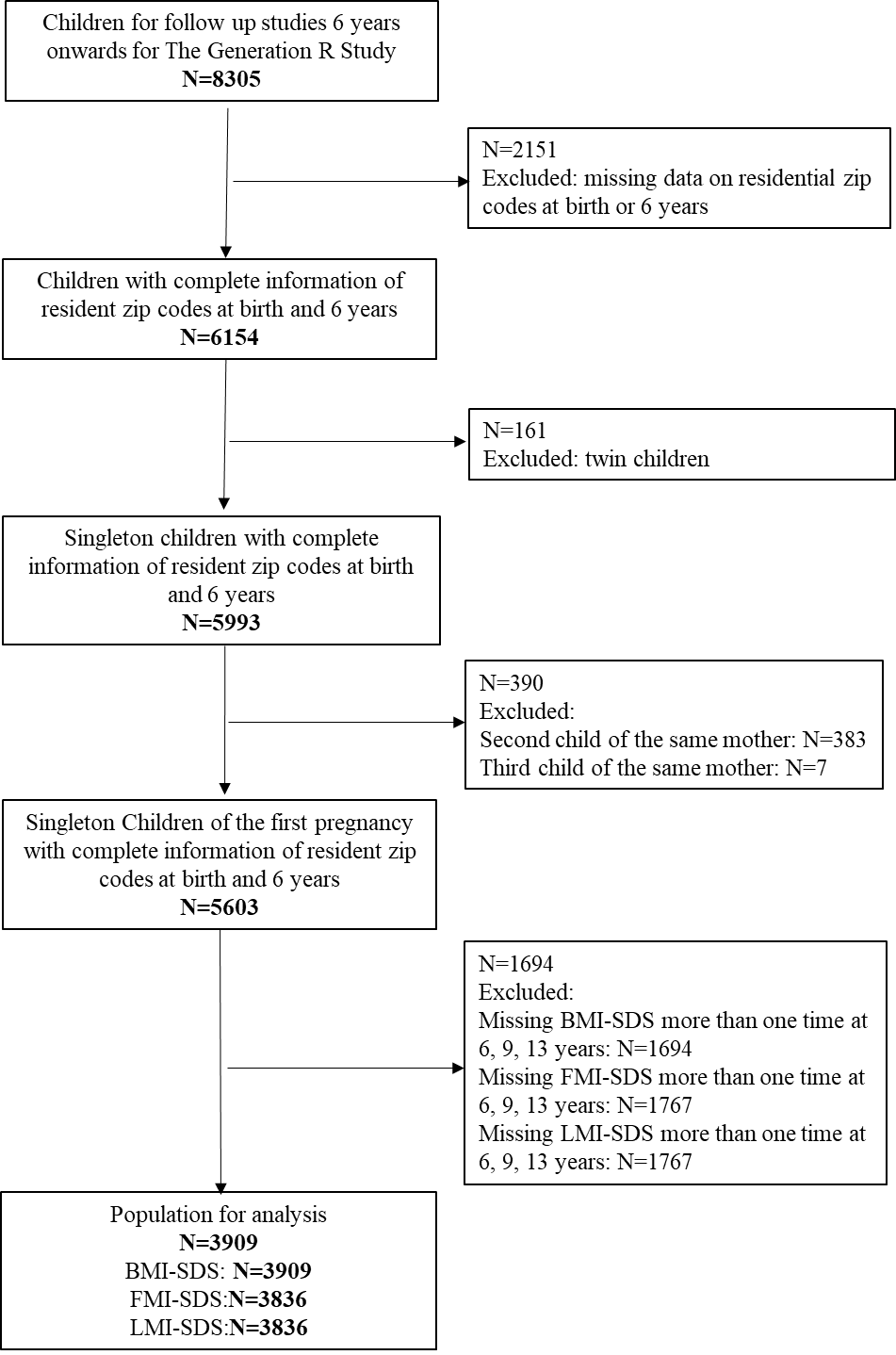
**

**Figure S1 Flowchart of the population in the study**

**
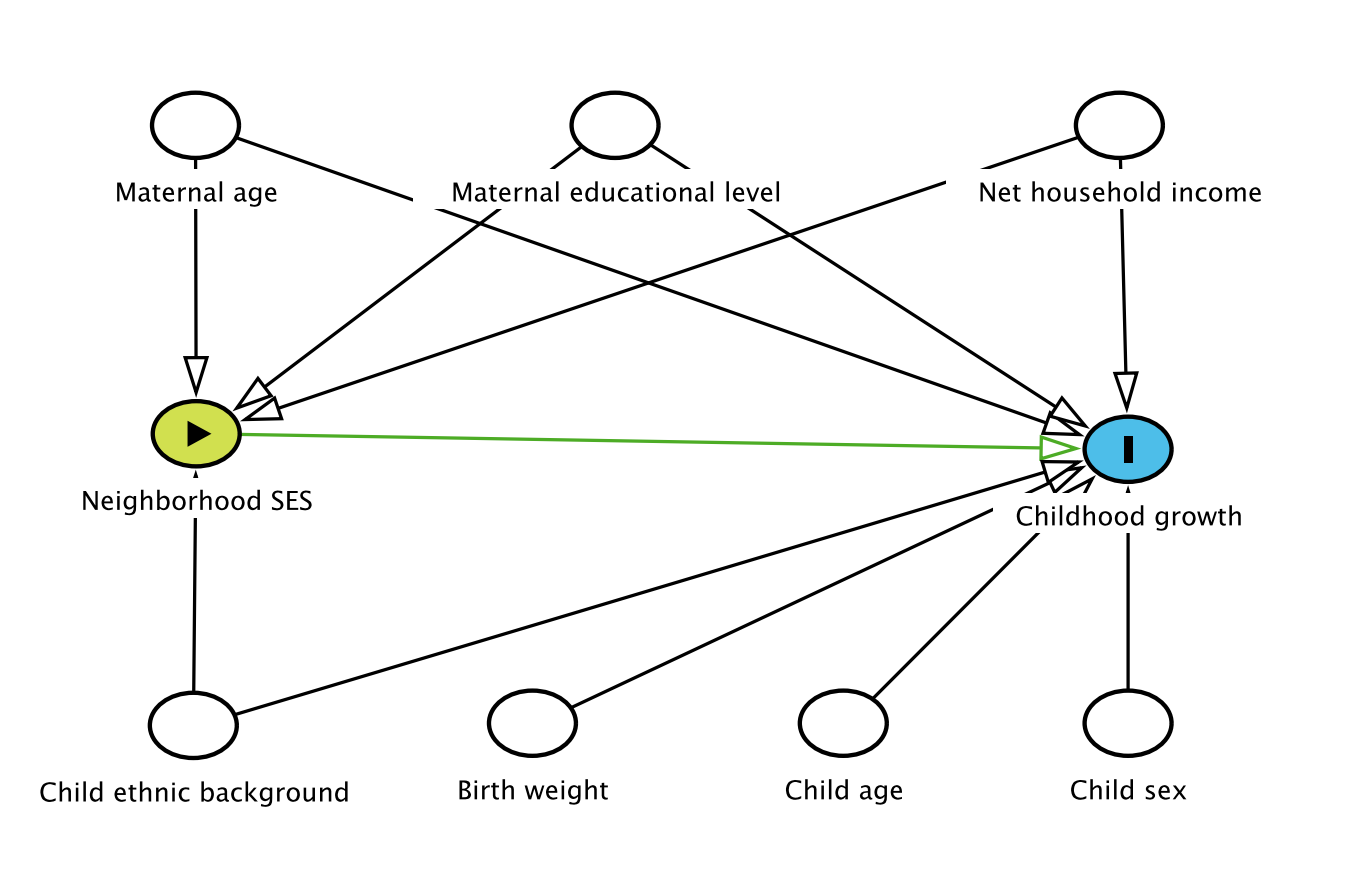
**

**Figure S2 Directed acyclic graph of assumed relationships between neighborhood SES and childhood growth based on literature data**

**Table S1. Characteristics of participants included and not included in the study (N=8305)**

|  | Total N=8305 | Not included N=4396 | Included N=3909 | P-value** |
| --- | --- | --- | --- | --- |
| **Family characteristics** |  |  |  |  |
| **Maternal age at enrollment (years), mean(SD)** | 30.28 (5.23) | 29.72 (5.22) | 30.91 (5.17) | **<0.001** |
| **Maternal education level, N(%)*** |  |  |  | **<0.001** |
| High | 3538 (56.6) | 1732 (59.4) | 1806 (54.2) |  |
| Low | 803 (12.8) | 355 (12.2) | 448 (13.4) |  |
| Middle | 1912 (30.6) | 831 (28.5) | 1081 (32.4) |  |
| **Net Household income, N(%)*** |  |  |  | **<0.001** |
| <2400 | 1804 (30.6) | 738 (27.0) | 1066 (33.7) |  |
| 2400~4000 | 2067 (35.1) | 952 (34.9) | 1115 (35.2) |  |
| ≥4000 | 2022 (34.3) | 1039 (38.1) | 983 (31.1) |  |
| **Child characteristics** |  |  |  |  |
| **Child ethnic background, N(%)*** |  |  |  | **<0.001** |
| Dutch | 4502 (56.5) | 2432 (58.5) | 2070 (54.3) |  |
| Other western | 677 (8.5) | 348 (8.4) | 329 (8.6) |  |
| Non-western | 2790 (35.0) | 1376 (33.1) | 1414 (37.1) |  |

Missing number: maternal age:3(0.4%), educational level: 2052(24.7%), household income: 2412(29.1%), ethnic background: 336(4.0%).

*The percentage for categorical variables is valid percentage.

**The t-test for continuous variables and chi-square test for categorical variables were used to test the differences between the included and not included.

**Table S2. Crude associations between the change in neighborhood SES and childhood weight status and body composition.**

|  | Boy | | | Girl | | | *P_interaction_** |
| --- | --- | --- | --- | --- | --- | --- | --- |
|  | n | β/RR (95%CI) | *P*-value | n | β/RR (95%CI) | *P*-value |  |
| **Weight status from 6 to 13 years old** | |  |  |  |  |  |  |
| **BMI-SDS** |  |  |  |  |  |  |  |
| Static-high group | 501 | Ref | Ref | 469 | Ref | Ref |  |
| Static-middle group | 323 | **0.17 (0.03, 0.31)** | **0.02** | 322 | **0.30 (0.16, 0.43)** | **<0.001** | 0.20 |
| Static-low group | 414 | **0.36 (0.23, 0.49)** | **<0.001** | 413 | **0.63 (0.50, 0.77)** | **<0.001** | **<0.01** |
| Upward mobility group | 377 | **0.15 (0.02, 0.28)** | **0.02** | 384 | **0.23 (0.11, 0.36)** | **<0.001** | 0.37 |
| Downward mobility group | 341 | **0.19 (0.06, 0.32)** | **0.01** | 365 | **0.16 (0.03, 0.29)** | **0.01** | 0.81 |
| **OWOB** |  |  |  |  | |  |  |
| Static-high group | 501 | Ref | Ref | 469 | Ref | Ref |  |
| Static-middle group | 323 | **1.99 (1.40, 2.83)** | **<0.001** | 322 | **2.73 (1.92, 3.90)** | **<0.001** | 0.22 |
| Static-low group | 414 | **2.37 (1.72, 3.27)** | **<0.001** | 413 | **4.99 (3.61, 6.90)** | **<0.001** | **<0.01** |
| Upward mobility group | 377 | **1.73 (1.23, 2.45)** | **<0.01** | 384 | **2.19 (1.54, 3.12)** | **<0.001** | 0.35 |
| Downward mobility group | 341 | **1.82 (1.28, 2.59)** | **<0.001** | 365 | **2.57 (1.82, 3.63)** | **<0.001** | 0.17 |
| **Body composition from 6 to 13 years old** | | |  |  |  |  |  |
| **FMI-SDS** |  |  |  |  |  |  |  |
| Static-high group | 492 | Ref | Ref | 458 | Ref | Ref |  |
| Static-middle group | 318 | **0.25 (0.12, 0.38)** | **<0.001** | 318 | **0.34 (0.23, 0.46)** | **<0.001** | 0.31 |
| Static-low group | 407 | **0.46 (0.34, 0.58)** | **<0.001** | 412 | **0.70 (0.57, 0.82)** | **<0.001** | **0.01** |
| Upward mobility group | 364 | **0.19 (0.07, 0.31)** | **<0.01** | 375 | **0.28 (0.17, 0.39)** | **<0.001** | 0.29 |
| Downward mobility group | 337 | **0.21 (0.09, 0.34)** | **<0.001** | 355 | **0.24 (0.13, 0.34)** | **<0.001** | 0.79 |
| **LMI-SDS** |  |  |  |  |  |  |  |
| Static-high group | 492 | Ref | Ref | 458 | Ref | Ref |  |
| Static-middle group | 318 | 0.03 (-0.09, 0.16) | 0.58 | 318 | **0.18 (0.05, 0.31)** | **0.01** | 0.10 |
| Static-low group | 407 | 0.10 (-0.01, 0.22) | 0.08 | 412 | **0.37 (0.24, 0.50)** | **<0.001** | **<0.01** |
| Upward mobility group | 364 | 0.07 (-0.05, 0.19) | 0.25 | 375 | 0.12 (0.00, 0.24) | 0.05 | 0.53 |
| Downward mobility group | 337 | 0.10 (-0.01, 0.21) | 0.08 | 355 | 0.07 (-0.06, 0.19) | 0.29 | 0.71 |

*Wald test was used to test the interaction effect of neighborhood SES mobility and child sex.

**Table S3. Associations between the change in neighborhood SES and childhood weight status and body composition stratified by relocation**

|  | Children relocated  (N=1534) | | | Children who did not move  (N=2375) | | |
| --- | --- | --- | --- | --- | --- | --- |
|  | N | β (95%CI) | *P*-value | N | β (95%CI) | *P*-value |
| **Weight status from 6 to 13 years old** |  |  |  |  |  |  |
| **BMI-SDS** |  |  |  |  |  |  |
| Static-high group | 264 | Ref | Ref | 706 | Ref | Ref |
| Static-middle group | 163 | -0.09 (-0.28, 0.09) | 0.31 | 482 | 0.09 (-0.02, 0.20) | 0.1 |
| Static-low group | 243 | 0.00 (-0.20, 0.19) | 0.99 | 584 | **0.20 (0.07, 0.33)** | **<0.01** |
| Upward mobility group | 526 | -0.04 (-0.18, 0.09) | 0.52 | 235 | 0.08 (-0.06, 0.22) | 0.24 |
| Downward mobility group | 338 | 0.02 (-0.12, 0.16) | 0.78 | 368 | 0.04 (-0.09, 0.16) | 0.57 |
| **OWOB** |  |  |  |  | |  |
| Static-high group | 264 | Ref | Ref | 706 | Ref | Ref |
| Static-middle group | 163 | 0.85 (0.50, 1.43) | 0.54 | 482 | **1.64 (1.20, 2.24)** | **<0.01** |
| Static-low group | 243 | 1.18 (0.72, 1.91) | 0.51 | 584 | **1.64 (1.18, 2.30)** | **<0.01** |
| Upward mobility group | 526 | 1.00 (0.66, 1.51) | 0.99 | 235 | 1.36 (0.91, 2.01) | 0.13 |
| Downward mobility group | 338 | 1.19 (0.77, 1.83) | 0.43 | 368 | **1.70 (1.22, 2.37)** | **<0.01** |
| **Body composition from 6 to 13 years old** | |  |  |  |  |  |
| **FMI-SDS** |  |  |  |  |  |  |
| Static-high group | 264 | Ref | Ref | 706 | Ref | Ref |
| Static-middle group | 163 | -0.06 (-0.21, 0.10) | 0.48 | 482 | **0.14 (0.04, 0.24)** | **0.01** |
| Static-low group | 243 | 0.07 (-0.11, 0.25) | 0.47 | 584 | **0.25 (0.13, 0.36)** | **<0.001** |
| Upward mobility group | 526 | -0.02 (-0.14, 0.11) | 0.79 | 235 | **0.13 (0.01, 0.26)** | **0.04** |
| Downward mobility group | 338 | 0.03 (-0.10, 0.16) | 0.69 | 368 | 0.09 (-0.02, 0.20) | 0.12 |
| **LMI-SDS** |  |  |  |  |  |  |
| Static-high group | 264 | Ref | Ref | 706 | Ref | Ref |
| Static-middle group | 163 | -0.05 (-0.23, 0.12) | 0.55 | 482 | 0.05 (-0.05, 0.15) | 0.32 |
| Static-low group | 243 | -0.05 (-0.23, 0.13) | 0.56 | 584 | 0.12 (-0.0002, 0.24) | 0.05 |
| Upward mobility group | 526 | 0.00 (-0.13, 0.13) | 0.96 | 235 | 0.00 (-0.14, 0.13) | 0.94 |
| Downward mobility group | 338 | 0.07 (-0.08, 0.21) | 0.36 | 368 | 0.00 (-0.11, 0.11) | 0.98 |

*In the model of BMI-SDS and OWOB, adjusted for maternal age, maternal educational level, family income, birth weight, sex, and ethnicity. In the model of FMI-SDS and LMI-SDS, child age was additionally adjusted.

**Table S4. Associations between the change in neighborhood SES between birth and 6 years and childhood weight status and body composition from 6 to 13 years.**

|  | Boy | | | Girl | | | *P_interaction_*** |
| --- | --- | --- | --- | --- | --- | --- | --- |
|  | n | β/RR (95%CI)* | *P*-value | n | β/RR (95%CI)* | *P*-value |  |
| **Weight status from 6 to 13 years old** |  |  |  |  |  |  |  |
| **BMI-SDS** |  |  |  |  |  |  |  |
| Static-high group | 501 | Ref | Ref | 469 | Ref | Ref |  |
| Static-middle group | 323 | -0.15 (-0.36, 0.06) | 0.16 | 322 | -0.05 (-0.24, 0.14) | 0.61 | 0.27 |
| Static-low group | 414 | -0.13 (-0.34, 0.09) | 0.25 | 413 | 0.09 (-0.12, 0.30) | 0.41 | **0.01** |
| Upward mobility group | 377 | -0.05 (-0.21, 0.10) | 0.50 | 384 | -0.05 (-0.20, 0.10) | 0.48 | 0.87 |
| Downward mobility group | 341 | 0.00 (-0.19, 0.19) | 0.99 | 365 | -0.08 (-0.27, 0.11) | 0.40 | 0.32 |
| **OWOB** |  |  |  |  | |  |  |
| Static-high group | 501 | Ref | Ref | 469 | Ref | Ref |  |
| Static-middle group | 323 | 1.00 (0.57, 1.77) | 0.99 | 322 | 1.17 (0.65, 2.10) | 0.60 | 0.42 |
| Static-low group | 414 | 0.86 (0.48, 1.52) | 0.60 | 413 | 1.50 (0.83, 2.70) | 0.18 | **<0.01** |
| Upward mobility group | 377 | 0.98 (0.64, 1.52) | 0.94 | 384 | 1.11 (0.67, 1.83) | 0.68 | 0.53 |
| Downward mobility group | 341 | 1.13 (0.67, 1.92) | 0.64 | 365 | 1.52 (0.86, 2.68) | 0.15 | 0.47 |
| **Body composition from 6 to 13 years old** | |  |  |  |  |  |  |
| **FMI-SDS** |  |  |  |  |  |  |  |
| Static-high group | 492 | Ref | Ref | 458 | Ref | Ref |  |
| Static-middle group | 318 | -0.06 (-0.25, 0.14) | 0.57 | 318 | -0.05 (-0.21, 0.11) | 0.55 | 0.47 |
| Static-low group | 407 | -0.01 (-0.21, 0.19) | 0.90 | 412 | 0.10 (-0.09, 0.28) | 0.31 | **0.02** |
| Upward mobility group | 364 | -0.03 (-0.16, 0.11) | 0.71 | 375 | -0.04 (-0.17, 0.10) | 0.60 | 0.64 |
| Downward mobility group | 337 | -0.01 (-0.19, 0.17) | 0.89 | 355 | -0.08 (-0.24, 0.08) | 0.34 | 0.53 |
| **LMI-SDS** |  |  |  |  |  |  |  |
| Static-high group | 492 | Ref | Ref | 458 | Ref | Ref |  |
| Static-middle group | 318 | -0.16 (-0.34, 0.03) | 0.10 | 318 | 0.06 (-0.13, 0.24) | 0.55 | 0.13 |
| Static-low group | 407 | -0.16 (-0.36, 0.03) | 0.10 | 412 | 0.12 (-0.08, 0.33) | 0.24 | **0.01** |
| Upward mobility group | 364 | -0.04 (-0.18, 0.10) | 0.59 | 375 | 0.01 (-0.13, 0.16) | 0.86 | 0.87 |
| Downward mobility group | 337 | 0.04 (-0.14, 0.21) | 0.69 | 355 | 0.07 (-0.12, 0.25) | 0.49 | 0.52 |

*In the model of BMI-SDS and OWOB, adjusted for maternal age, maternal educational level, family income, birth weight, ethnicity, and the change in neighborhood SES between 6 to 13 years. In the model of FMI-SDS and LMI-SDS, child age was additionally adjusted.

**Wald test was used to test the interaction effect of neighborhood SES mobility and child sex.

**Table S5. Associations between the change in neighborhood SES between birth and 6 years and childhood weight status and body composition from 6 to 13 years.**

|  | Boy | | | Girl | | | *P_interaction_*** |
| --- | --- | --- | --- | --- | --- | --- | --- |
|  | n | β/RR (95%CI)* | *P*-value | n | β/RR (95%CI)* | *P*-value |  |
| **Weight status from 6 to 13 years old** |  |  |  |  |  |  |  |
| **BMI-SDS** |  |  |  |  |  |  |  |
| Static-high group | 501 | Ref | Ref | 469 | Ref | Ref |  |
| Static-middle group | 323 | -0.02 (-0.15, 0.12) | 0.82 | 322 | 0.08 (-0.06, 0.22) | 0.25 | 0.22 |
| Static-low group | 414 | 0.05 (-0.10, 0.20) | 0.51 | 413 | **0.26 (0.10, 0.42)** | **<0.01** | **0.01** |
| Upward mobility group | 377 | 0.02 (-0.11, 0.15) | 0.79 | 384 | 0.01 (-0.12, 0.14) | 0.84 | 0.79 |
| Downward mobility group | 341 | 0.13 (0.00, 0.26) | 0.05 | 365 | 0.01 (-0.13, 0.14) | 0.94 | 0.24 |
| **OWOB** |  |  |  |  | |  |  |
| Static-high group | 501 | Ref | Ref | 469 | Ref | Ref |  |
| Static-middle group | 323 | 1.22 (0.83, 1.80) | 0.31 | 322 | **1.44 (0.97, 2.14)** | **0.07** | 0.41 |
| Static-low group | 414 | 1.12 (0.74, 1.68) | 0.60 | 413 | **1.97 (1.32, 2.94)** | **<0.01** | **0.01** |
| Upward mobility group | 377 | 1.10 (0.76, 1.61) | 0.60 | 384 | 1.24 (0.84, 1.83) | 0.29 | 0.48 |
| Downward mobility group | 341 | **1.49 (1.02, 2.18)** | **0.04** | 365 | **1.65 (1.13, 2.40)** | **0.01** | 0.54 |
| **Body composition from 6 to 13 years old** | |  |  |  |  |  |  |
| **FMI-SDS** |  |  |  |  |  |  |  |
| Static-high group | 492 | Ref | Ref | 458 | Ref | Ref |  |
| Static-middle group | 318 | 0.06 (-0.07, 0.18) | 0.36 | 318 | 0.11 (-0.01, 0.22) | 0.08 | 0.38 |
| Static-low group | 407 | 0.13 (-0.01, 0.27) | 0.07 | 412 | **0.27 (0.13, 0.41)** | **<0.01** | **0.04** |
| Upward mobility group | 364 | 0.05 (-0.07, 0.16) | 0.45 | 375 | 0.05 (-0.06, 0.16) | 0.39 | 0.63 |
| Downward mobility group | 337 | **0.13 (0.00, 0.25)** | **0.04** | 355 | 0.05 (-0.06, 0.16) | 0.40 | 0.44 |
| **LMI-SDS** |  |  |  |  |  |  |  |
| Static-high group | 492 | Ref | Ref | 458 | Ref | Ref |  |
| Static-middle group | 318 | -0.07 (-0.19, 0.06) | 0.28 | 318 | 0.08 (-0.05, 0.21) | 0.22 | 0.10 |
| Static-low group | 407 | -0.02 (-0.16, 0.11) | 0.73 | 412 | **0.20 (0.05, 0.35)** | **0.01** | **0.01** |
| Upward mobility group | 364 | 0.00 (-0.13, 0.12) | 0.94 | 375 | 0.03 (-0.10, 0.15) | 0.69 | 0.71 |
| Downward mobility group | 337 | 0.10 (-0.02, 0.21) | 0.11 | 355 | 0.03 (-0.10, 0.16) | 0.68 | 0.40 |

*In the model of BMI-SDS and OWOB, adjusted for maternal age, maternal educational level, family income, birth weight, ethnicity, and children’s psychiatric symptoms. In the model of FMI-SDS and LMI-SDS, child age was additionally adjusted.

**Wald test was used to test the interaction effect of neighborhood SES mobility and child sex.
